# Cortical Thinning in Healthy Aging Correlates with Larger Motor-Evoked EEG Desynchronization

**DOI:** 10.3389/fnagi.2016.00063

**Published:** 2016-03-29

**Authors:** David Provencher, Marie Hennebelle, Stephen C. Cunnane, Yves Bérubé-Lauzière, Kevin Whittingstall

**Affiliations:** ^1^Department of Nuclear Medicine and Radiobiology, Université de SherbrookeSherbrooke, QC, Canada; ^2^Research Center on Aging, Université de SherbrookeSherbrooke, QC, Canada; ^3^Department of Medicine, Université de SherbrookeSherbrooke, QC, Canada; ^4^Department of Pharmacology and Physiology, Université de SherbrookeSherbrooke, QC, Canada; ^5^Department of Electrical and Computer Engineering, Université de SherbrookeSherbrooke, QC, Canada; ^6^Sherbrooke Molecular Imaging Center, Université de SherbrookeSherbrooke, QC, Canada; ^7^Department of Diagnostic Radiology, Université de SherbrookeSherbrooke, QC, Canada

**Keywords:** EEG, aging, cortical thickness, cortical depth, event-related desynchronization

## Abstract

Although electroencephalography (EEG) is a valuable tool to investigate neural activity in patients and controls, exactly how local anatomy impacts the measured signal remains unclear. Better characterizing this relationship is important to improve the understanding of how inter-subject differences in the EEG signal are related to neural activity. We hypothesized that cortical structure might affect event-related desynchronization (ERD) in EEG. Since aging is a well-documented cause of cortical thinning, we investigated the effects of cortical thickness (CT) and cortical depth (CD – the skull-to-cortex distance) on ERD using anatomical MRI and motor-evoked EEG in 17 healthy young adults and 20 healthy older persons. Results showed a significant negative correlation between ERD and CT, but no consistent relationship between ERD and CD. A thinner cortex was associated with a larger ERD in the α/β band and correcting for CT removed most of the inter-group difference in ERD. This indicates that differences in neural activity might not be the primary cause for the observed aging-related differences in ERD, at least in the motor cortex. Further, it emphasizes the importance of considering conditions affecting the EEG signal, such as cortical anatomical changes due to aging, when interpreting differences between healthy controls and/or patients.

## Introduction

Electroencephalography (EEG) acquires integrated measures of brain electrical activity, but how the signal arises within the brain is still poorly understood. A better understanding of the origin of the EEG signal may help explain the differences in EEG signal strength observed between different brain areas in a single individual (Cvetkovic and Cosic, 2009) and/or between the same areas across individuals or groups (Armitage et al., 2000). For instance, Rossiter et al. (2014) have shown that motor-evoked magnetoencephalography (MEG) modulation is stronger in healthy older compared to young adults. On the other hand, similar studies using hemodynamic measures have reported the opposite effect: BOLD task-activation is weaker in healthy older relative to young adults (Riecker et al., 2006; Roski et al., 2014). One possibility for this discrepancy is that age-related changes in cortical structure have different effects on neural compared to hemodynamic activity. We previously showed that inter-region and inter-individual differences in cerebral venous structure affect the BOLD signal (Vigneau-Roy et al., 2013), so the age-related decline in cerebrovascular function (Kalaria, 1996) may contribute to the age-related decrease in BOLD. However, the impact of the vascular and cortical structural changes occurring with age on EEG remains unclear.

Electroencephalography measures an electric potential which decreases as 1/*r*^2^ (*r* being the distance between the electrode on the scalp and the cortical source of neural activity) for dipoles (Nunez and Srinivasan, 2005) and as 1/*r* for monopoles (Riera et al., 2012). Therefore, increasing the physical distance between the skin and the surface of the cortex, i.e., CD, would also be expected to decrease EEG amplitude. Indeed, Frodl et al. (2001) observed a moderate negative correlation between the P300 amplitude and temporo-parietal skull thickness, though no such correlation was observed in the frontal lobe. Hagemann et al. (2008) also observed some modest negative correlations between resting α power (8–12 Hz) and skull thickness, depending on electrode location. These findings suggest that differences in skull thickness may partially explain differences in EEG amplitude, but neither of these studies reported CD.

Electroencephalography is believed to be primarily driven by synchronous synaptic potentials of pyramidal cells oriented perpendicular to the cortical surface, forming current dipoles (Nunez and Srinivasan, 2005). Temporal synchrony across this mass of neurons is a particularly important component of the EEG signal because strong, albeit asynchronous, activity will cancel out at the macroscopic scale, yielding smaller EEG signals at the surface of the scalp (Musall et al., 2014). Although the origin of synaptic synchrony is debated (Ecker et al., 2010), its range may be limited by anatomical constraints. For instance, the aging brain displays reduced CT (Westlye et al., 2009; Thambisetty et al., 2010; Gaetz et al., 2012; van Velsen et al., 2013; Nugent et al., 2014), particularly in areas near the motor cortex (Salat et al., 2004; Koo et al., 2012). Age-related cortical thinning seems not to change the absolute number of neurons in the cortex (Peters et al., 1998; Peters and Sethares, 2002), resulting in increased neuron density with age (Leuba and Garey, 1987). Since the density of connections between neurons declines with increasing separation of their cell bodies (Braitenberg and Schüz, 1991; Wright and Bourke, 2013), increasing neuron density (i.e., decreasing CT) might actually increase synchrony (Maheswaranathan et al., 2012) and thus increase EEG amplitude.

In the present study, we sought to evaluate the effects of CD and CT on EEG event-related desynchronization (ERD), i.e., the decrease in oscillatory power during a task with respect to the baseline (Pfurtscheller and Lopes da Silva, 1999). Assessing this relationship is important given that ERD measures are often used in clinical studies investigating Parkinson’s disease (Heida et al., 2014) and epilepsy (Krause et al., 2008). We therefore collected motor-evoked EEG data in two separate age groups – 17 healthy young adults and 20 cognitively normal older adults, allowing us to investigate the same brain area (motor cortex) in two populations with different CT and possibly CD due to a wide difference in age. We found a significant negative correlation between ERD and CT, but no consistent relationship between ERD and CD between groups.

## Materials and Methods

### Experimental Design

To investigate variations of ERD due to differences in CT and/or CD, this study design aimed to obtain distinct datasets with as broad a range in cortical anatomical parameters as possible. Therefore, we recruited younger and older adults, who would be expected to display significant differences in CT and possibly CD due to brain atrophy with age. Both groups performed two similar motor tasks, namely left and right FT, which should activate similar regions of the brain with little intra-group anatomical variations. We then located the sources of EEG activity to confine the CD and CT estimation to regions of the brain contributing to the measured ERD signal.

### Participants

The two age groups comprised of 17 healthy young adults (aged 22.8 ± 2.6 years, 14 right-handed) and 20 healthy older adults (aged 74.2 ± 5.9 years, 17 right-handed) and were matched for participant handedness distribution (82 and 85% right-handed, respectively) to obtain comparable FT results. Each participant underwent MRI and EEG acquisitions within a 1 year period. The study was conducted in accordance with the ethics committees of the Centre Hospitalier Universitaire de Sherbrooke and the Centre de Santé et des Services Sociaux – Institut Universitaire de Gériatrie de Sherbrooke. Each participant gave written informed consent prior to participation.

### MRI Acquisition and Preprocessing

Anatomical T1-weighted MR images were acquired for each participant on a 1.5 Tesla Magnetom Symphony scanner (Siemens, Germany) using an MPRAGE sequence (TR/TE 1860/3.54 ms, 1 mm isotropic) for the younger group and using a gradient-echo sequence (TR/TE 1600/4.68 ms, 1 mm isotropic) for the older group. Although different sequences were used, the T1 images were qualitatively similar in both groups. Image processing included non-local means denoising using DIPY (Garyfallidis et al., 2014), computing subject-specific gray matter masks and CT measures using FreeSurfer (Reuter et al., 2012) as well as registration to the ICBM 2009c nonlinear asymmetric template in MNI space (Fonov et al., 2011) using ANTs (Avants et al., 2011).

### Cortical Depth Maps

**FIGURE 1:**
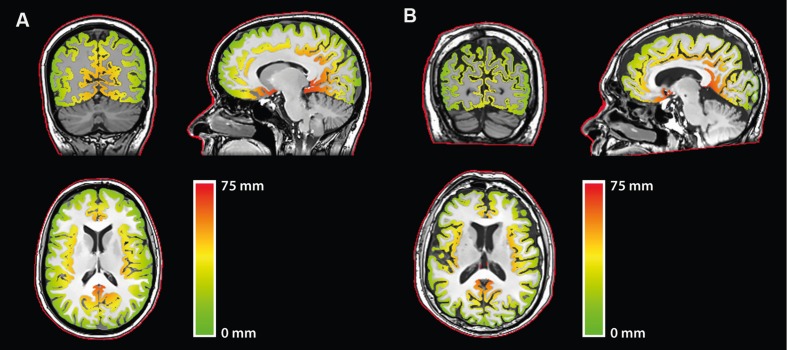
**Cortical depth (CD) maps relative to the scalp for representative **(A)** younger and **(B)** older participants**. *Red outline:* Scalp outline used in the computation.

To compute CD, here defined as the distance from the scalp to the surface of the cortex, first a mask of the head of each participant was obtained by thresholding the anatomical T1 image. The outline of the scalp was then obtained through erosion and subtraction of the head masks. Computing the shortest distance from each voxel of the gray matter mask to the scalp outline yielded CD maps (**Figure 1**).

### EEG Acquisition and Preprocessing

EEG was acquired using a 64 channel actiCAP Ag/AgCl system and BrainAmp MR plus amplifiers (BrainProducts GmbH, Germany) at 250 Hz with electrode positions following the 10–20 system, using FCz as reference. Tasks were performed in a block design using auditory cues starting with a 30 s rest period, followed by five repetitions of 20 s task and 30 s rest epochs. Each participant performed sequential FT tasks with their left (FTL) and right (FTR) hands with eyes closed. Participants were instructed to perform the FT sequence as quickly as possible without errors. These tasks were selected to produce desynchronization in the α and β bands, since α activity is predominant in awake subjects at rest with eyes closed (Laufs et al., 2003) and β band power decreases during motor tasks (Ritter et al., 2009).

Electroencephalography signal processing was performed using the EEGLAB toolbox (Delorme and Makeig, 2004). Channels around the ears (FT9, FT10, T7, T8, TP9, TP10, PO9, and PO10) were removed in all participants due to excessive noise levels. Up to two additional bad channels were removed and interpolated in three younger and six older participants. The remaining EEG channels were bandpass-filtered from 0.1 to 45 Hz to remove low-frequency signal drifts (a necessary step for independent component analysis – ICA), as well as higher frequency noise (to reduce EEG source localization variability). For computational purposes, EEG data were down-sampled to 128 Hz. ICA was then computed and artefactual components were rejected through visual inspection. EEG data were then re-referenced to the common average.

### EEG Power and ERD

The power spectral density (PSD) of EEG signals at each electrode was computed independently for each rest and task epoch. Average rest and task power were computed by integrating the PSDs over the α/β band (7.5–25 Hz) and averaging across epochs. Subject-specific ERDs were obtained based on (Pfurtscheller and Lopes da Silva, 1999) by computing the mean percent change in power during the task epochs compared to the rest (baseline) epochs and averaging the results across the electrodes of interest (CPz, CP1, CP2, CP3, CP4, Cz, C1, C2, C3, and C4). These electrodes were selected due to their proximity to the motor cortex. Baseline power and ERD were then compared across groups for each task, with a more negative ERD value representing a larger desynchronization. To ensure that results were not affected by fortuitous electrode selection, the average across all electrodes was also computed as a control.

### EEG Source Localization

Cortical sources of EEG activity in the frequency range of interest (7.5–25 Hz) were localized for each individual task and rest epochs using sLORETA (Pascual-Marqui, 2002), which is well-suited for the localization of distributed sources of activity without *a priori* knowledge of their number. This yielded sources in 6239 5 mm × 5 mm × 5mm gray-matter cortical voxels in MNI space.

### Activation Regions of Interest (ROIs)

We defined regions of interest (ROI) based on EEG activation to determine ‘local’ measures of CT and CD in the activated brain regions producing the ERD signal. In order to reduce dependence on source localization spatial accuracy while confining the activation ROIs to anatomically relevant regions in each participant, we aimed to produce ROIs using group-averages of activation that were spatially diffuse, yet confined in cortical gray matter. Group-average sLORETA modulation maps were therefore obtained by computing the percent change of mean source current densities during all task epochs with respect to rest epochs in each sLORETA voxel of individual participants, and then averaging results within each group. From these group maps, we computed activation ROIs in each participant through upsampling to a 1 mm isotropic grid, registration to anatomical T1 space, thresholding (removing modulation values below the 85th percentile), clustering (removing clusters with less than 30 voxels) and masking voxels outside gray matter. Additionally, we performed controls to further ensure that results would not depend solely on source localization accuracy and ROI selection. First, we used each group’s sLORETA modulation map to produce ‘matched’ activation ROIs in the other group to allow comparison of similar ROIs in the two groups. Second, we used all gray-matter voxels in both groups to yield ‘whole-cortex’ measures.

The mean CT and CD over the activation ROIs were computed in each participant and results were compared between the two groups and task conditions using two-tailed two-sample *t*-tests. The analysis was then repeated with the ‘matched’ ROIs as a control. We assessed ERD correlations with CT and CD using both Pearson’s correlation coefficient (r) and Spearman’s rank correlation coefficient (ρ) to reduce outlier sensitivity and avoid misinterpretation of results that can arise with single correlation metric (Hauke and Kossowski, 2011; Rousselet and Pernet, 2012; Schwarzkopf et al., 2012) as well as identify linear or non-linear relationships. For correlation computations, absolute ERD values were used to reflect that more negative ERDs imply larger desynchronization. This was repeated with controls, i.e., using ‘matched ROIs’ or using all electrodes and ‘whole-cortex’ CT and CD measures. Significance testing was performed with a threshold of 5% using false discovery rate (FDR) correction for multiple comparisons (α_FDR_ = 0.05). Except where otherwise indicated, MRI image processing was performed using AFNI (Cox, 1996) and EEG signal processing was performed using MATLAB (The Mathworks, USA).

## Results

### Group-Average ERD Maps

**FIGURE 2:**
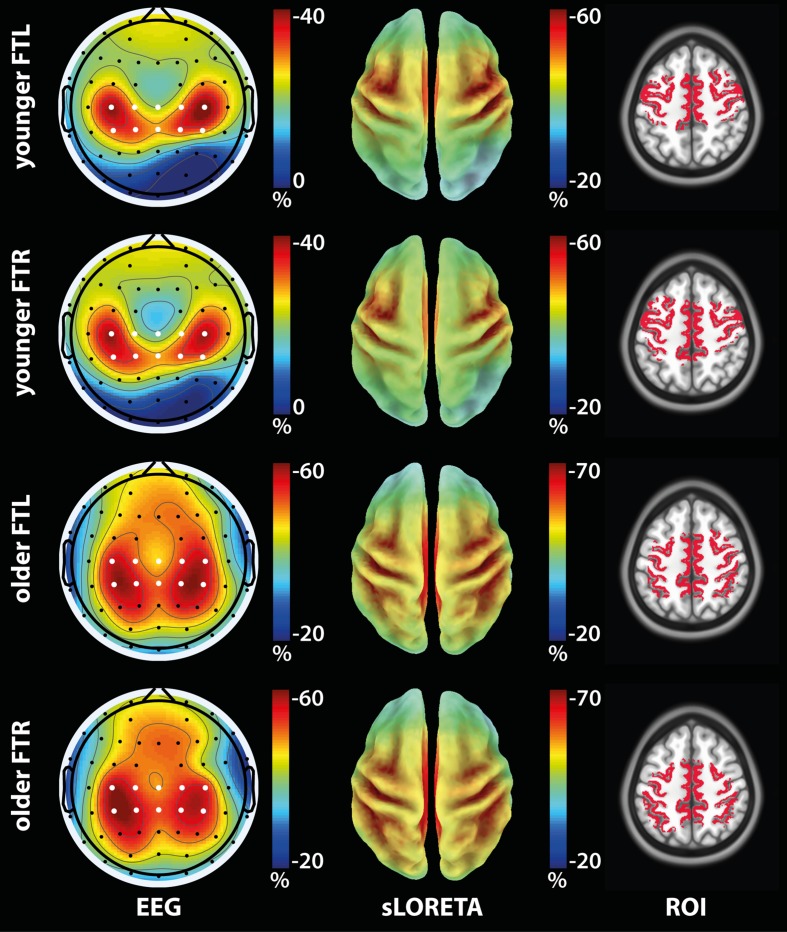
**Activation ROI definition from ERD maps in the α/β band (7.5–25 Hz) for the older and younger groups for the FTL and FTL conditions**. **(Left)**: Group-average ERD topographic maps. *White dots:* Electrodes used in the ERD computation. **(Center)**: Group-average sLORETA modulation maps. **(Right)**: Activation ROIs shown on the anatomical template.

Group-average topographic maps of ERD (**Figure 2**, left column) were consistent with the motor nature of the task and displayed bilateral symmetry (Pfurtscheller and Lopes da Silva, 1999). Maximum ERD values were similar across FTL and FTR conditions, but were greater for the older compared to the younger group.

### Activation ROIs

The group-average sLORETA modulation maps (**Figure 2**, center column) were qualitatively consistent with the ERD maps in terms of both spatial distribution and relative maximum value across tasks. The associated group-activation ROIs (**Figure 2**, right column) obtained in subject space included the main regions expected to be solicited by the tasks, i.e., the primary motor cortex for both the FTL and FTR conditions. Further, ROIs were mostly symmetrical about the midline and displayed greater spatial extent in the contralateral hemisphere. Moreover, activation ROIs associated with FTL and FTR partially overlapped within each group, but were more posterior in the older compared to the younger group.

### FTL vs. FTR in the Younger and Older Groups

In both age groups, no significant differences (*p* > 0.05, FDR-corrected) were observed between the FTL and FTR conditions (Supplementary Figure S1) in terms of ERD, baseline power and CT in the activation ROI. Although significant, the difference in CD between the FTL and FTR ROIs was small for the younger group. In the older group, no such difference in CD was observed. Data from the FTL and FTR conditions were therefore pooled, effectively doubling the number of data points in each group for the FT condition in further analyses.

### FT in the Younger vs. Older Groups

**FIGURE 3:**
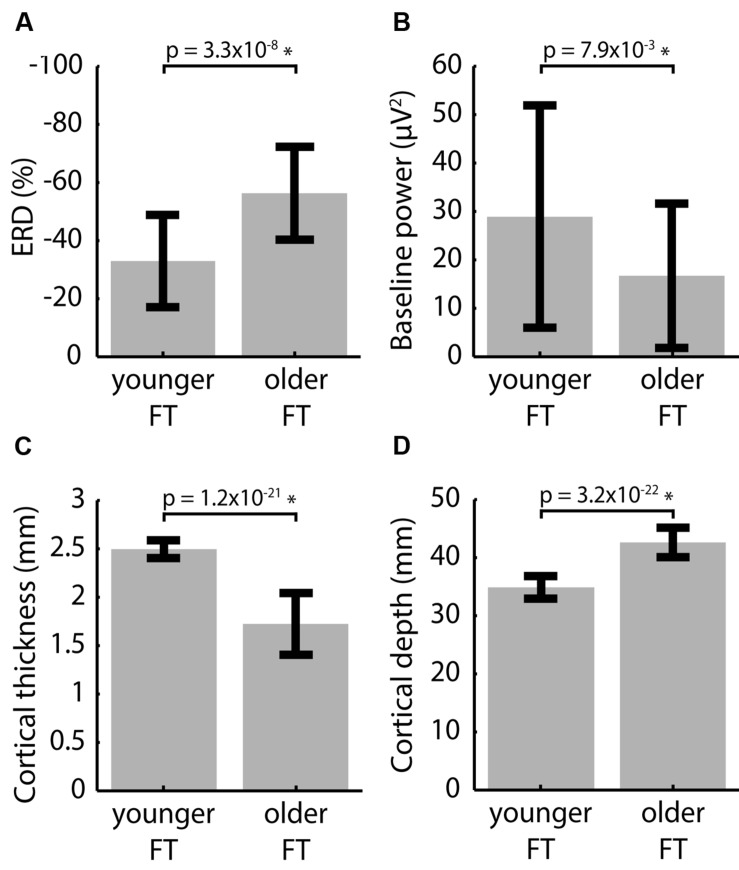
**Inter-group differences between pooled left and right FT data for **(A)** ERD, **(B)** baseline power, **(C)** CT and **(D)** CD averaged over the activation ROIs**. *p*-values of two-tailed two-sample *t*-tests are indicated. ^∗^indicates significance at the α_FDR_ = 0.05 level.

Compared to the younger group, the older group displayed smaller baseline power and larger ERD in the FT conditions and the cortex was both thinner and deeper in the associated ROIs (*p* < 0.05 FDR corrected, see **Figure 3**). Using all electrodes did not change the conclusions for baseline power and ERD (Supplementary Figure S2). Performing controls using the ‘matched’ and ‘whole-cortex’ measures (Supplementary Figure S3) also did not alter the findings for CT, but the differences in CD between the two groups essentially disappeared. These findings show that regardless of cortical location baseline power was lower, ERD was consistently stronger and the cortex was consistently thinner in the older group. On the other hand, CD results were inconsistent, i.e., the effect observed in the main analysis did not manifest in any of the controls.

**FIGURE 4:**
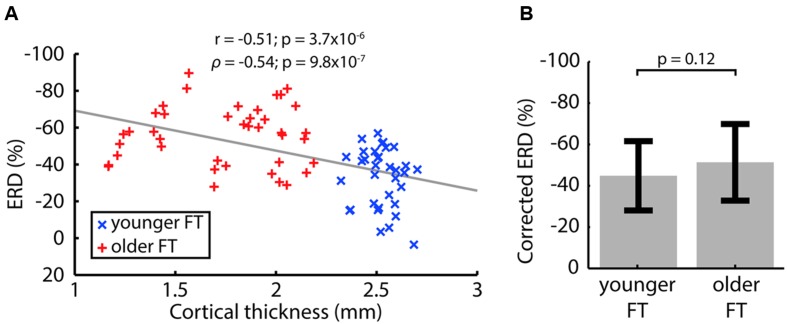
**(A)** Event-related desynchronization (ERD) correlation with CT for pooled left and right FT data of the younger and older groups. Pearson’s correlation coefficient (r) and Spearman’s rank correlation coefficient (ρ) are indicated with associated *p*-values. Correlations were computed using absolute ERD values to reflect that a more negative ERD implies a larger desynchronization. **(B)** ERD corrected for CT. The *p*-value of a two-tailed two-sample *t*-test is indicated.

Since between-group differences in CT but not CD were consistent across controls, we investigated the relationship between ERD and CT, but no statistically significant within-group correlation was observed. However, pooling data from the younger and older groups revealed a significant negative correlation in which a thinner cortex was associated with larger ERD (**Figure 4A**). This correlation was consistently present in controls (using ‘matched’ ROIs; using all electrodes as well as ‘whole cortex’ ROIs). The fact that r and ρ were similar suggested a linear relationship between ERD and CT, allowing the use of linear regression. After regressing out the effect of CT, the difference in ERD between younger and older participants became smaller and not statistical significant (**Figure 4B**). On the other hand, no correlation was found between baseline power and ERD (*r* = 0.098, *p* = 0.41; ρ = 0.072, *p* = 0.54).

To ensure that results were not driven by outliers, we performed an additional control by repeating the analysis while excluding seven participants who displayed whole-brain CT values outside the range expected from the literature (Supplementary Figure S4). Doing so did not significantly alter the results (see Supplementary Table S1).

## Discussion

The main finding of this study is that the age-related increase in motor-induced ERD was significantly negatively correlated with CT, but CD was ROI-dependent and did not display an obvious relationship with ERD. After correcting for CT, ERD differences between the older and the young group disappeared, suggesting that age-related differences in ERD may not reflect differences in neural activity *per se*, but rather differences in some other aspect of cortical anatomy such as CT.

### EEG vs. CT

The significant negative association between ERD and CT was consistently present (i) when measuring CT in group-specific ROIs (**Figures 3A,C**) and ‘matched’ control ROIs when using task-appropriate electrodes to measure ERD (Supplementary Figures S1A,B,E,F), as well as (ii) in ‘whole-cortex’ ROIs when using all electrodes (Supplementary Figures S2A and S3A–C). Nevertheless, no statistically significant inter-individual correlation was found between ERD and CT, which is consistent with previous results in visual evoked potential studies (Elvsåshagen et al., 2015). This might be attributed to the relatively small inter-individual variations relative to the noise in the CT measurement. Indeed, CT is ultimately derived from a 1 mm anatomical image, whereas within-group standard deviations in CT were 0.092 and 0.32 mm for the younger FT and older FT datasets, respectively. On the other hand, the span of CT values across groups (1.35–2.77 mm) was consistent with the anatomical image resolution and group differences in CT were robust to controls, which might explain why a correlation was observed between ERD and CT when data from both age groups were pooled. In any case, the difference in ERD between the younger and older groups was greatly diminished when regressing out the effect of CT (**Figure 4B**). This demonstrates the importance of taking confounding factors, such as cortical anatomical structure, into account when comparing patient and/or healthy control data, rather than directly interpreting stronger EEG modulation as larger amplitude or extent of activation.

Although anatomical MRI images were acquired using different sequences for each group, the whole-cortex CT average of most participants relative to their age (Supplementary Figure S4) was in good agreement with the literature for similar age ranges (Salat et al., 2004; Lemaitre et al., 2012). However, seven older participants displayed CT values lower than expected for their age (1.4–1.8 mm, compared to 2.0–2.4 mm typical values). We could not determine whether this reflected actual physiology or methodological differences; therefore whether these data points were outliers remains unclear. Nevertheless, all participants (younger and older) were cognitively normal. Performing the analysis with or without these seven subjects gave similar results and identical conclusions (Supplementary Table S1). In both cases, the between-group difference in CT (0.69 mm for all participants; 0.49 mm with the seven low values removed) was within the expected range given the age difference (Salat et al., 2004; Lemaitre et al., 2012; van Velsen et al., 2013; Zhou et al., 2013; Fjell et al., 2014). We interpret these results to support the main findings of this study, i.e., that ERD differences observed in aging are related to CT and should not be attributed to differences in neural activity alone.

### CD Differences between the Younger and Older Groups

Cortical depth was significantly larger in the older group when using group-specific ROIs (**Figure 3D**), but the difference disappeared when using ‘matched’ ROIs derived from either group’s average sLORETA modulation maps or when using whole-brain metrics (Supplementary Figures S3D–F). Whereas aging-related loss of white and gray matter volume (Lemaitre et al., 2012; Nugent et al., 2014) as well as increase in cerebrospinal fluid volume (Courchesne et al., 2000; Good et al., 2001) would suggest increased CD across the brain with age, we observed no aging-related difference in CD between groups across the whole brain (Supplementary Figure S3F). We believe that this can be explained by a small change in CD or ventricle expansion in the older group. The difference observed in the main analysis was therefore due to ROI position and spatial extent, rather than to increased CD in the older group. As such, we deemed the CD metric overly dependent on exact activation localization to rely on EEG-derived activation ROI and did not compute CD-ERD and CD-CT relationships, because they could be misleading. In fact, average CD measures obviously strongly depend on spatial localization and extent (e.g., an ROI 1 mm deeper will have an average CD 1 mm larger), more so than CT which varies slowly. This can explain why ROI-average CT results were robust to controls, contrary to CD. Robust assessment of CD-ERD and CD-CT relationships thus requires more precise activation ROI identification through other means than EEG source localization and is therefore left for future study.

### Effect of Anatomy on ERD

Since ERD computation implies dividing task signals by rest signals, observing a significant correlation between anatomy and ERD is somewhat counterintuitive because the static effects would be expected to cancel out. A possible explanation could be that local electrical fields generated by neurons might spatially and temporally interfere in a different manner during the rest versus task periods, resulting in an anatomy-dependent modulation of the EEG signal. Further, the fact that no correlation was found between baseline rest power and ERD supports the hypothesis that the age-related increase in neuron density (due to lower CT and stable number of neurons) increases synchrony, and therefore EEG amplitude, implying larger desynchronization effects during a task. Still, computing modulation of EEG signals acquired in a single session should benefit from canceling out the effects of electrode impedance, static effects (e.g., age) and the basal state of the participant (i.e., alertness, caffeine intake, etc.). For instance, α/β baseline power was lower in the older group in accordance with the literature (Voytek et al., 2015), but the opposite was true of ERD.

### Group-Average vs. Subject-Specific Activation ROIs

To study co-variations in anatomy and ERD, anatomical parameter estimation would ideally be confined to subject-specific activated regions. Using subject-specific sLORETA modulation maps to produce activation ROIs could help achieve this goal, but the results would become more dependent on homogeneity of EEG data quality, denoising efficacy and source localization accuracy across participants. To avoid this, we used group-average sLORETA modulation maps to define activation ROIs with a large spatial extent, since they provide spatially smooth source estimates. Nevertheless, since EEG source localization is an ill-posed inverse problem, control ROIs were used for validation to avoid conclusions relying solely on specific ROI selection. In future studies, using EEG-fMRI acquisitions to produce more robust subject-specific or group average BOLD activation ROIs could avoid this issue.

## Conclusion

In conclusion, ERD in the α/β band was significantly negatively correlated with CT (but not with CD) in motor-evoked EEG data in young and cognitively normal older persons. Cortical thinning due to aging was associated with increased ERD and correcting for CT removed most of the inter-group variability in ERD. When computing EEG modulation or ERD, potential differences in CT should therefore be taken into account when interpreting signal differences between participants.

## Author Contributions

The authors contributed to this manuscript in the following manner: study design (DP, SC, KW), data acquisition and analysis (DP, MH), interpretation of results (DP, SC, YBL, KW). All authors contributed to revise and approve the final version of the manuscript and agree to be accountable for this work.

## Conflict of Interest Statement

The authors declare that the research was conducted in the absence of any commercial or financial relationships that could be construed as a potential conflict of interest.

## ABBREVIATIONS

CD: Cortical depth
CT: Cortical thickness
FT: Finger tapping
FTL: Finger tapping with the left hand
FTR: Finger tapping with the right hand
