# Supplementary material for: Cortical Thinning in Healthy Aging Correlates with Larger Motor-Evoked EEG Desynchronization
Source: Front Aging Neurosci. 2016 Mar 29;8:63. doi: 10.3389/fnagi.2016.00063 (PMC4809888; doi:10.3389/fnagi.2016.00063)
Supplement: Supplementary file 2 [file Image_1.PDF]

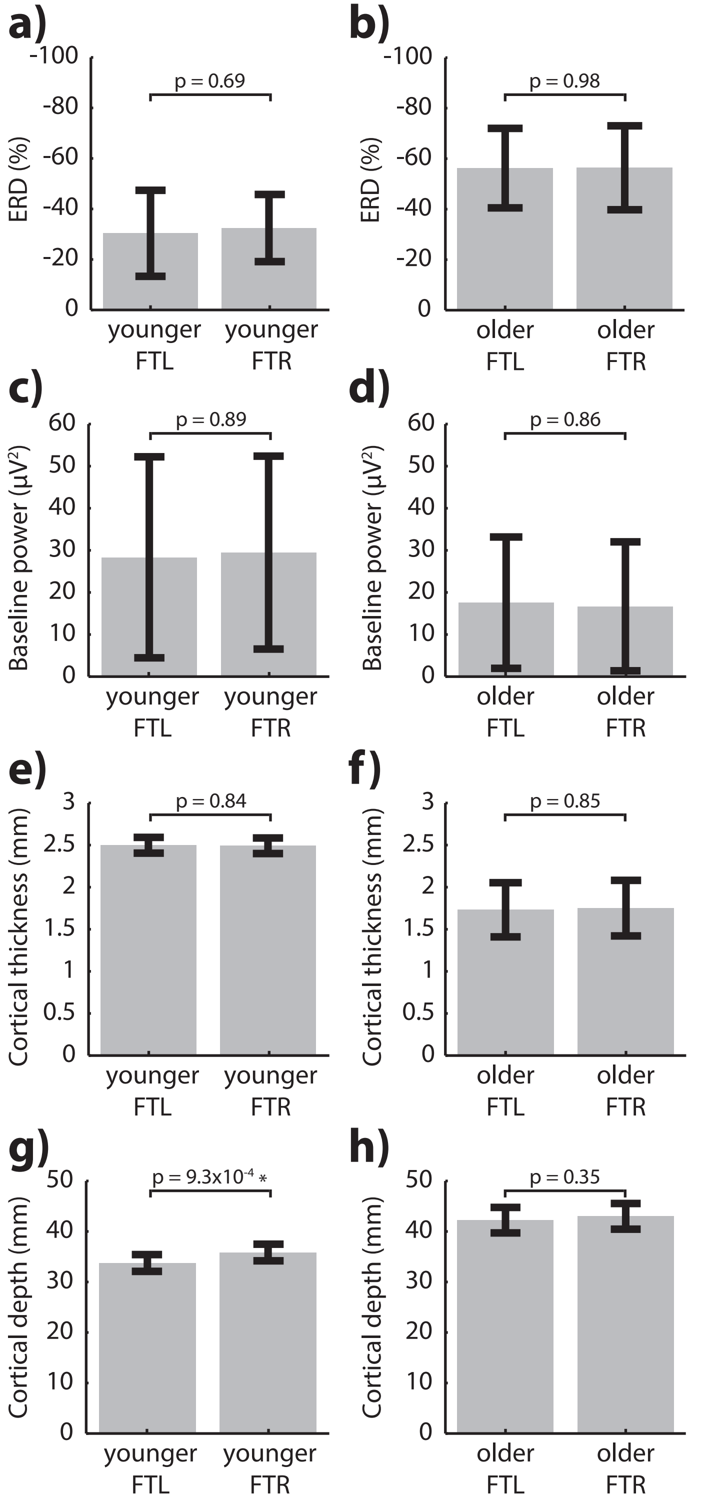


**Figure S1:** Intra-group differences between left and right finger tapping conditions in terms of **a)**, **b)** alpha/beta event-related desynchronization and **c)**, **d)** baseline power as well as **e)**, **f)** cortical thickness and **g)**, **h)** cortical depth averaged over the activation ROIs. *Left:* younger group. *Right:* older group. *p values of two-tailed two-sample t-tests are indicated. * indicates significance at the* $a_{FDR} = 0.05$ *level.*
