# Supplementary material for: Cortical Thinning in Healthy Aging Correlates with Larger Motor-Evoked EEG Desynchronization
Source: Front Aging Neurosci. 2016 Mar 29;8:63. doi: 10.3389/fnagi.2016.00063 (PMC4809888; doi:10.3389/fnagi.2016.00063)
Supplement: Supplementary file 3 [file Image_2.PDF]

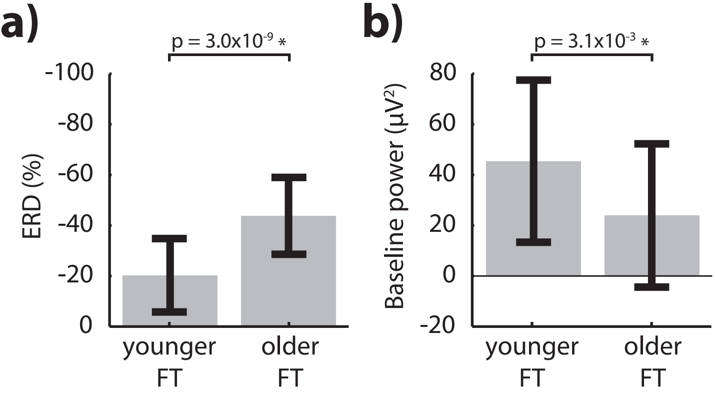

Figure S2: Inter-group comparison of **a)** ERD and **b)** baseline power computed using all electrodes and pooled left and right finger tapping data. *p values of two-tailed two-sample t-tests are indicated. * indicates significance at the* $a_{FDR} = 0.05$ *level.*
