# Supplementary material for: Cortical Thinning in Healthy Aging Correlates with Larger Motor-Evoked EEG Desynchronization
Source: Front Aging Neurosci. 2016 Mar 29;8:63. doi: 10.3389/fnagi.2016.00063 (PMC4809888; doi:10.3389/fnagi.2016.00063)
Supplement: Supplementary file 4 [file Image_3.PDF]

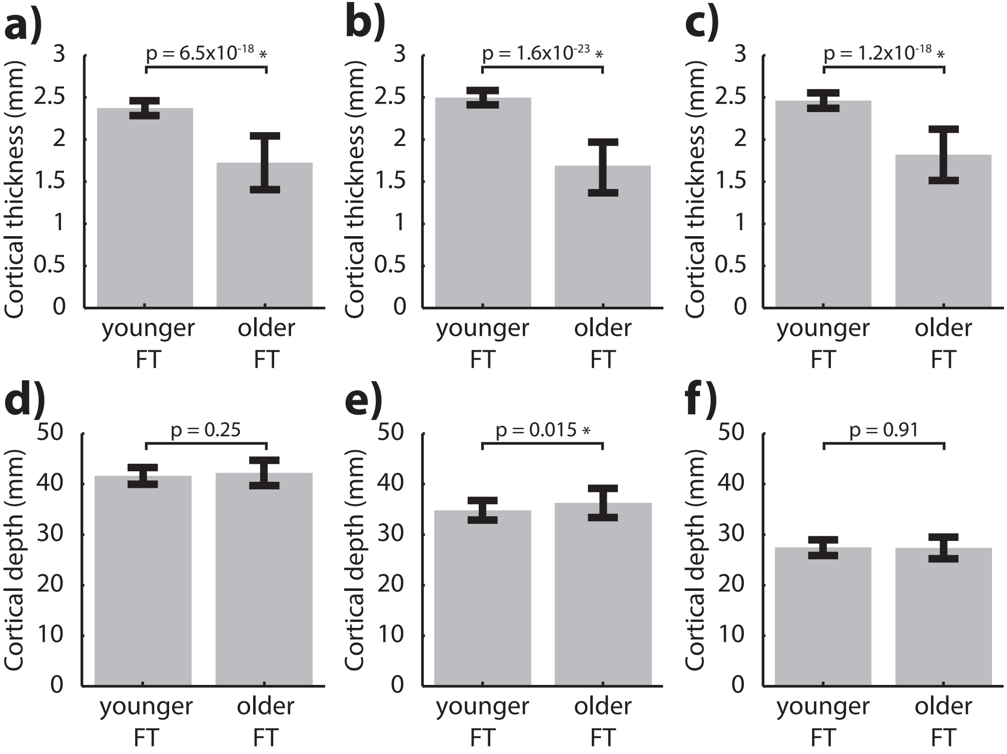

Figure S3: Inter-group differences for the pooled left and right finger tapping data in terms of **a)-c)** cortical thickness and **d)-f)** cortical depth averaged over control ROIs. *Left:* Using the older group's ROIs in both groups. *Center:* Using the younger group's ROIs in both groups. *Right:* Using the 'whole-cortex' ROI. *p values of two-tailed two-sample t-tests are indicated. * indicates significance at the* $a_{FDR} = 0.05$ *level.*
