# Supplementary figures and images for: Cortical Thinning in Healthy Aging Correlates with Larger Motor-Evoked EEG Desynchronization
Source: Front Aging Neurosci. 2016 Mar 29;8:63. doi: 10.3389/fnagi.2016.00063 (PMC4809888; doi:10.3389/fnagi.2016.00063)

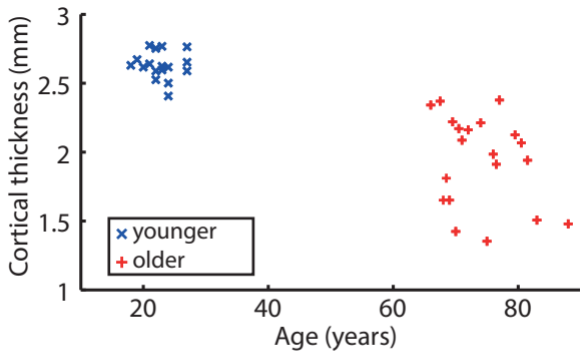

**Figure S4:** Average CT over the whole cortex versus age for all participants.

Supplement: Supplementary file 5 [file Image_4.PDF]
